# Unobtrusive Sensors for Synchronous Monitoring of Different Breathing Parameters in Care Environments

**DOI:** 10.3390/s24072233

**Published:** 2024-03-31

**Authors:** Imran Saied, Aaesha Alzaabi, Tughrul Arslan

**Affiliations:** 1 Advanced Care Research Centre, The University of Edinburgh, Edinburgh EH9 3JW, UK; isaied@ed.ac.uk; 2 School of Engineering, The University of Edinburgh, Edinburgh EH9 3JW, UK; a.h.m.alzaabi@sms.ed.ac.uk

**Keywords:** antennas, noninvasive monitoring, respiratory rate, ultra-wideband, Wi-Fi

## Abstract

Respiratory problems are common amongst older people. The rapid increase in the ageing population has led to a need for developing technologies that can monitor such conditions unobtrusively. This paper presents a novel study that investigates Wi-Fi and ultra-wideband (UWB) antenna sensors to simultaneously monitor two different breathing parameters: respiratory rate, and exhaled breath. Experiments were carried out with two subjects undergoing three breathing cases in breaths per minute (BPM): (1) slow breathing (12 BPM), (2) moderate breathing (20 BPM), and (3) fast breathing (28 BPM). Respiratory rates were captured by Wi-Fi sensors, and the data were processed to extract the respiration rates and compared with a metronome that controlled the subjects’ breathing. On the other hand, exhaled breath data were captured by a UWB antenna using a vector network analyser (VNA). Corresponding reflection coefficient data (S11) were obtained from the subjects at the time of exhalation and compared with S11 in free space. The exhaled breath data from the UWB antenna were compared with relative humidity, which was measured with a digital psychrometer during the breathing exercises to determine whether a correlation existed between the exhaled breath’s water vapour content and recorded S11 data. Finally, captured respiratory rate and exhaled breath data from the antenna sensors were compared to determine whether a correlation existed between the two parameters. The results showed that the antenna sensors were capable of capturing both parameters simultaneously. However, it was found that the two parameters were uncorrelated and independent of one another.

## 1. Introduction

Advances in science and medicine have helped to increase the longevity of people’s lifespans. Unfortunately, this comes with a problem that poses a new global and socioeconomic threat: a rapid increase in the ageing population. In 2020, the number of people aged 60 and older reached approximately 1 billion, outnumbering children younger than 5 years old [1]. By 2050, the world’s population of people aged 60 years and older will double to 2.1 billion. While this trend started in high-income countries, it is now low- and middle-income countries that are experiencing the most significant change. As a result, by 2050, two-thirds of the world’s population over 60 years of age will live in low- and middle-income countries [1]. It is therefore of paramount importance that care-based and health monitoring technologies are investigated and implemented in the living environments of the ageing population (e.g., care homes, independent homes). This will ensure that timely care is provided when needed.

A common problem among the ageing population is the onset of breathing- and respiratory-related problems [2]. As with other organs, the effects of ageing on the respiratory system cause a gradual decline in its maximum functionality. Age-related changes in the lungs include the following [3]:

- Decreases in peak airflow (how quickly someone can exhale) and the exchange of carbon dioxide and oxygen.
- Decreases in measures of lung function such as vital capacity (the maximum amount of air that can be breathed out following a maximal inhalation).
- Weakening of the respiratory muscles.
- Decline in the effectiveness of lung defence mechanisms.

Therefore, it is essential to have sensors or devices that help monitor a person’s respiration rate (RR) and identify early signs of potential health issues through respiratory abnormalities. 

Currently, several techniques are used to assess an individual’s general respiratory health. The most common methods are pulmonary function tests (PFTs), which include spirometry, which can be used to assess a patient’s airflow, and full-body plethysmography, used to assess lung volumes [4,5,6]. While these evaluations effectively assess a patient’s respiratory health at a specific time in a laboratory setting, they cannot continuously monitor a patient’s respiratory state under normal daily environments. Continuous respiration monitoring can be achieved through various methods, such as (1) respiratory inductive plethysmography (RIP), which uses two inductive belts placed around the abdomen and ribcage to measure the changes in circumference during respiration, (2) optoelectronic plethysmography, which measures the motion of the chest wall and abdomen visually using cameras or depth sensors, and (3) transthoracic impedance, which calculates RR and air volume by measuring the change in impedance of the torso between several electrodes during respiration [7,8,9,10]. While these methods can all accurately track RR and air volume, they are either cumbersome to wear or require constant line-of-sight access to the patient’s entire torso, limiting their use to research or clinical settings. 

Several wearable respiration monitors have been developed that are small, discrete, and easy for patients to wear [11,12,13,14]. However, none of these wearable sensors reported in the literature measure hydration in relation to RR, which serves as the motivation for this study. Researchers in [4] developed a wearable unobtrusive sensor capable of measuring both RR and tidal volume, which is the amount of air moving out of the lungs. However, like all wearable sensors, they still rely on its placement on a person’s body, which, for older people, may be uncomfortable and, therefore, obtrusive.

Recently, several studies have investigated the use of antennas as an unobtrusive way of monitoring breathing. Specifically, RR is determined by measuring phase changes introduced by the target to the transmission signal. Several radar systems have been proposed in the literature for detecting vital signs. These include continuous-wave (CW) [15] and ultra-wideband (UWB) [16] radars. Among CW radars, some studies have looked at single-tone [17], frequency-modulated continuous-wave (FMCW) [18], and stepped frequency-modulated continuous-wave (SFCW) [19] radars for RR monitoring. 

While these studies showed promising results in accuracy and sensitivity, they all focused on validating antennas to sense only one breathing parameter: RR. Hence, they did not investigate additional parameters related to breathing, such as tidal volume or water volume during exhalation. However, researchers have investigated RR with other non-breathing-related parameters such as heart rate and sleep [20,21]. 

One possible parameter that could be investigated with antennas is the dielectric changes due to exhaled air. Studies have shown that as a person exhales air, a large amount of water vapour is exhaled, compared to the air inhaled [22]. This is due to the moisture inside the mouth and lungs that is also pushed out as gas when air is exhaled. Exhaled air contains as much as 91% relative humidity (RH) compared to inhaled air [23]. As a result of this increase in water content, this would lead, theoretically, to an increase in the dielectric properties (e.g., relative permittivity) of the air surrounding the mouth when a person exhales. 

Several studies have looked at the investigation of wearable or on-body RF antennas to measure RH, especially in the ultra-high-frequency (UHF) range [24,25,26,27]. The study conducted in [27] provided interesting analysis of an antenna’s performance in changing RH levels. It was found that there was a linear relationship between resonant frequency and RH, where the resonant frequency decreased as RH levels increased. However, all of these studies looked at the measurement of RH in static conditions, whereas RH during exhalation is a dynamic process. To the best of our knowledge, no work has investigated the use of antennas to detect dielectric changes in a person’s exhaled breath as a parameter to monitor breathing. In addition to this, no studies exist that have investigated this parameter along with RR sensing from chest movements to monitor the quality of a person’s breathing.

In this study, a novel experiment was conducted that utilised two sensors to unobtrusively and noninvasively monitor a person’s breathing based on two different breathing parameters: (1) RR, and (2) dielectric changes due to exhaled air. A Wi-Fi sensing system was used to capture chest movements to measure RR, while a UWB antenna was fabricated and used for measuring the water vapour emitted from a person’s breath at exhalation. The Wi-Fi sensor was used to measure the RR as an independent variable for this experiment, while the humidity in the exhaled air was measured as a result of varying RR, offering a completely unobtrusive solution for measuring these aspects. As this study serves as a proof-of-concept, the experiments were conducted with two subjects (i.e., the authors: one male and one female). Both antennas were used to capture data concerning the different parameters (i.e., chest movements and exhaled breath). In particular, channel state information (CSI) data were captured based on chest movements, while S11 data were captured for exhaled air. Additionally, conventional devices and programs were used to compare and validate measurements obtained from the antenna sensors.

## 2. Materials and Methods

Since two different breathing parameters were investigated together in this study, it was essential that the right antennas and sensors be designed and used accordingly. First, the respiratory rate can be distinguished based on the channel state information at the Wi-Fi receiver. Furthermore, Wi-Fi sensors are affordable and widely available. For these reasons, Wi-Fi sensors were selected in order to monitor the respiratory rates unobtrusively. For the second parameter, exhaled breaths, it is essential to look at dielectric changes in the area in front of the mouth. This is because when a person exhales, there is a higher percentage of water vapour due to fluid in the lungs, airway, and mouth, which is blown out along with the exhaled air [22]. An important parameter that can be used to measure the amount of water vapour in the exhaled breath is relative humidity (RH). By definition, RH is simply defined as the ratio of how much water vapour is in the air to how much water vapour the air could potentially contain at a given temperature [28]. It varies with the temperature of the air, where colder air can hold less vapour. Therefore, after exhalation, the air would have a higher dielectric value, which would affect reflected signals from an antenna. For this purpose, a UWB antenna was chosen and modified to detect exhaled breaths from a distance unobtrusively. 

### 2.1. Wi-Fi Sensor for Detecting Respiratory Rates

Fundamentally, CSI is collected through Wi-Fi communication as a part of the channel estimation procedure in the IEEE 802.11n protocol. CSI describes the signal propagation through the channel between the transmitter and receiver, effectively describing the environment as well as the dynamic changes in the channel, including movement. Access to the CSI data enables a more sensitive Wi-Fi sensing across a larger sensing range. This is in contrast to previous works that used the received signal strength indicator (RSSI), which often depends on proximity to line of sight (LOS) [29]. 

#### 2.1.1. Wi-Fi CSI Sensing Background

CSI corresponds to the measurements of the channel frequency response (CFR) across each subcarrier in an orthogonal frequency-division multiplexing (OFDM) system, such as

(1) $$H\left(f,t\right)=\sum_{i=1}^{K}H_{i}\left(f,t\right)=\sum_{i=1}^{K}\begin{array}{c} \alpha_{i}\;\left(t\right)\;e^{-j2\pi f\tau_{i}\left(t\right)} \end{array}$$

where *K* corresponds to the number of multipaths, $H_{i}\left(f,t\right)$ is the channel response of the *i*-th path at time *t*, *α*(*t*) is the amplitude attenuation factor affected by changes in the surrounding environment, and *τ(t)* is the propagation delay. *H*(*f,t*) is the CSI matrix considering that *y*(*t*) *= H*(*f,t*)*x(*t*) *+ n*(*t*), where *x*(*t*) is the transmitted signal, *y*(*t*) is the received signal, and *n*(*t*) is the noise. Subsequently, the *N* CSI measured for all 52 subcarriers can be represented as follows:

(2) $$\;\;\;H\left(i\right)=\left[H_{1\;}\left(i\right)\;H_{2}\left(i\right),\;\ldots ,\;H_{52}\left(i\right)\right],\;\;\;\;\;\;where\;i=1,\;2,\;\ldots ,\;N\;$$

where $H_{k}\left(i\right)$ contains the amplitude and the phase information, such as

(3) $$H_{i}\left(f,t\right)=\left|H_{i}\left(f,t\right)\right|+e^{j∠H_{i}\left(f,t\right)}$$

The signal processing procedure relies on the variations imposed by movement on CSI amplitude, where principal component analysis (PCA) is applied after filtering the frequencies of interest, as in our previous work [30].

#### 2.1.2. Wi-Fi CSI Sensing Hardware

Despite the exciting applications introduced by Wi-Fi sensing, most consumer hardware does not support access to CSI information without hardware tampering. This can be attributed mostly to Wi-Fi technology being primarily used for communication, while sensing lies outside the scope of typical Wi-Fi applications. Nevertheless, some devices on the market offer access to CSI data through developed software tools. For instance, the Intel 5300 Wi-Fi Network Interface Card (NIC) provides access to 30 Wi-Fi CSI subcarriers through the Linux 802.11n CSI tool [31]. In addition, some Atheros NICs support the extraction of CSI data for all 52 subcarriers using the Atheros CSI tool [32]. However, the ESP32 microcontrollers offer direct access to all 52 Wi-Fi subcarriers’ CSI data and can offer standalone operation, reducing cost and creating the prospect for realistic multi-sensor implementation [33]. 

**Figure 1:**
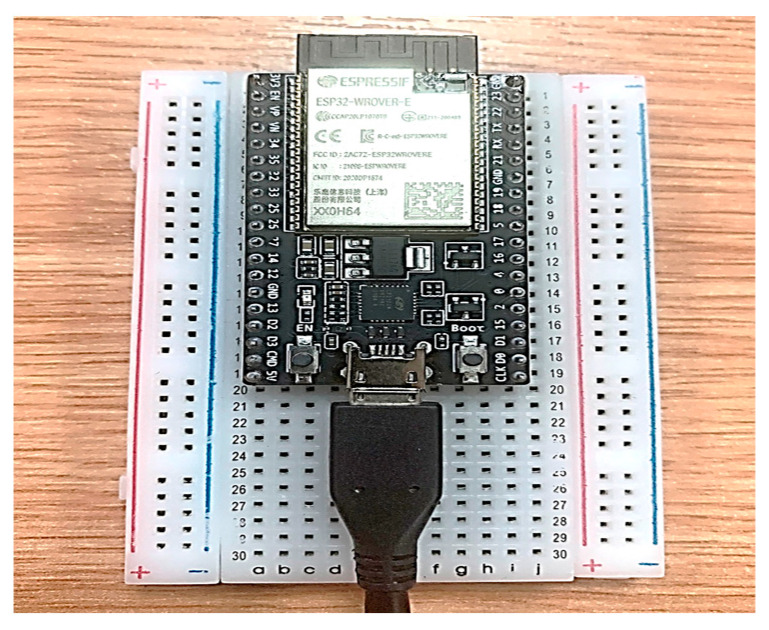
ESP32 microcontroller development kit placed onto a breadboard.

For our work, two units of the ESP32-DevKitC-VE (as shown in Figure 1) were used as Wi-Fi sensors, where one acted as a transmitter (TX) and the other as a receiver (RX). The development kit includes a built-in PCB omnidirectional antenna operating in the 2.4 GHz band, which is used to transmit and receive Wi-Fi packets for sensing purposes.

### 2.2. Ultra-Wideband Antenna for Detecting Exhaled Breaths

Ultra-wideband (UWB) antennas have been used extensively for medical applications in the last 10 to 15 years. Fundamentally, UWB antennas that are used for medical applications focus on capturing changes in dielectric properties to determine the presence of an illness or disease (e.g., cancer, Alzheimer’s disease, etc.) [34]. Dielectric properties include a relative permittivity and conductivity component that indicates how well electric current (and, in the case of UWB antennas, electromagnetic waves) can freely travel in the object [35]. Dielectric properties are closely related to the amount of water in the object, where an object with more water content would have a higher conductivity value and a lower permittivity value, and vice versa [35]. 

In the case of this study, this fundamental principle of UWB sensing can be applied to exhaled water vapour detection by having a sensor monitor the area around the mouth. The dielectric properties of this area will consistently change as a person breathes. Specifically, during the time of inhalation, there is an intake of air and water vapour, which will lead to an instance of lower dielectric properties. However, at exhalation, there is a large volume of air and water vapour that is exhaled out of the mouth, which then causes an instance of a sudden increase in dielectric properties. It is through this process of exhalation that a UWB antenna can be useful to capture another breathing parameter to determine a person’s quality of breathing.

Unlike conventional antennas that operate within a narrow frequency band, UWB antennas can operate across a wide frequency range, typically from a few hundred megahertz (MHz) to several gigahertz (GHz) [19]. This wide bandwidth allows for the transmission of signals with high data rates and enables precise localisation of targets. In the case of this study, the wide bandwidth allowed the emitted RF signals to travel and reach the location of a person breathing to capture the exhaled water vapour. In addition, UWB antennas can operate at relatively low power levels, making them safer for medical applications and non-ionising compared to high-power electromagnetic radiation sources.

The antenna used for detecting the exhaled breaths was a modified ultra-wideband antenna (UWB) originally published in [36]. The antenna is a trapezoidal-shaped planar monopole antenna that exhibits ultra-wideband performance. A planar monopole antenna was chosen due to its low-profile configuration as compared to the Vivaldi antenna, which has a high-profile structure along the direction of propagation, making it inconvenient and not ideal as an unobtrusive antenna. In general, a monopole antenna is made of two main parts: a feeding line and a radiator. The optimisation of both would lead to optimal bandwidth. While the trapezoidal-shaped patch was selected as the radiating element, the feeding line used a co-planar waveguide (CPW) structure. 

**Figure 2:**
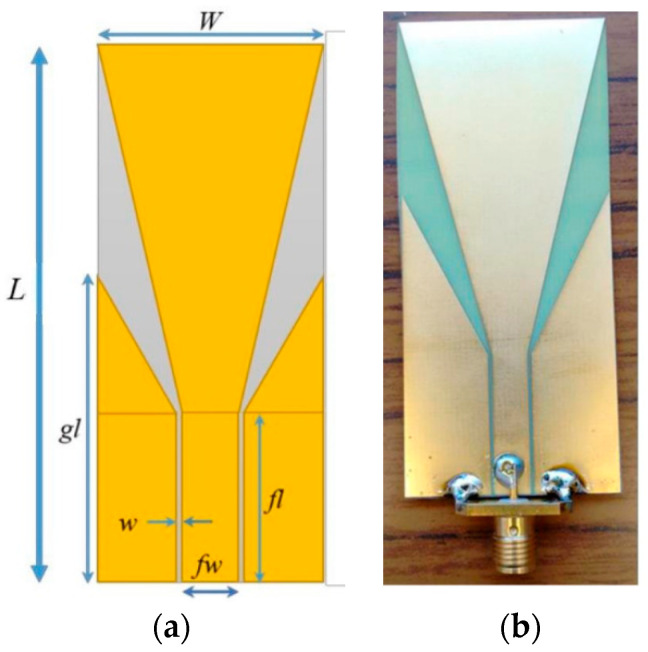
(**a**) Geometry of antenna, and (**b**) screenshot of fabricated antenna.

Since the previous design of the antenna in [36] was applied for stroke detection, further analysis and design modifications had to be carried out in CST Studio Suite to ensure that the propagated RF waves from the antenna could reach the area of interest (i.e., a person’s mouth). Therefore, a frequency band between 0.75 GHz and 3 GHz was utilised. Based on this frequency range, and validated by CST simulations, the UWB antenna could propagate RF waves to a subject between 30 cm and 2.33 m. However, it should be noted that the further the RF waves propagate, the weaker they become, therefore leading to a higher chance of them not being reflected. In addition, because we utilised Wi-Fi-based sensors along with UWB antenna sensors, it was important to ensure that the right distance between the subject and UWB sensor was chosen, such that the quality of the RF waves was not drastically affected by noise, and allowing for the RF signals to reach the area of interest and reflect back efficiently. While the shape of the antenna elements remained the same, the overall dimensions of the antenna needed to be changed, since the antenna now had to detect a person’s exhaled breath from a distance unobtrusively. The final design of the UWB monopole antenna is shown in Figure 2a, while the parameters of the antenna are given in Table 1.

The substrate of the antenna that was used was a 0.2 mm thick FR-4 substrate. The FR-4 material is commonly used for PCBs. This material was chosen due to its strong structure, high insulation, minimal interference, and water resistance. Because the antenna was to be placed away from the person, this material ensured that it would be sturdy and could remain in place. The antenna was fabricated using photolithography technique. The metallisation was only on one side of the substrate. An SMA connecter was then soldered onto the antenna. Figure 2b shows the fabricated UWB antenna that was used for detecting exhaled breaths.

### 2.3. Experiment Setup

**Figure 3:**
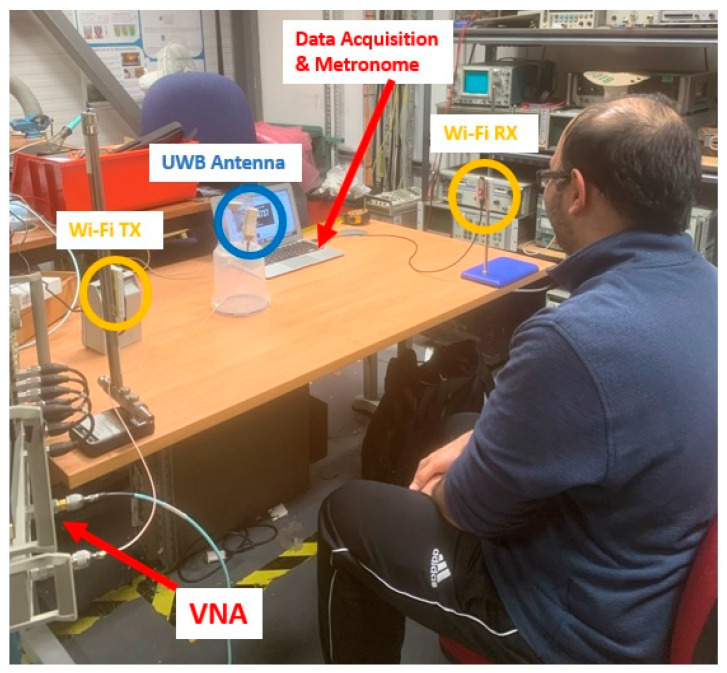
Experimental setup showing the position of the Wi-Fi receiver/transmitter antennas (labelled Wi-Fi RX and Wi-Fi TX, respectively) to capture RR data, along with the UWB antenna for capturing exhaled breath data.

The system setup used for the experiments is shown in Figure 3. The experimental setup consisted of a PC, vector network analyser (VNA), Wi-Fi sensors (receiver and transmitter), and UWB antenna. 

The experiments were conducted inside a lab with two subjects: one male and one female. The subjects underwent three different breathing scenarios with the following RR in breaths per minute (BPM): (1) 12 BPM (low), (2) 20 BPM (middle), and (3) 28 BPM (high). A metronome was used to follow the subjects’ breathing rates accordingly and served as the ground truth to compare the processed RR from the Wi-Fi sensors. The subjects underwent the breathing exercise for 2 min for each breathing scenario. During that time, the following data were recorded for each case: (1) continuous data from the Wi-Fi sensors, along with individual timestamps; (2) 10 scans (taken at 10-second intervals) from the UWB antenna via the VNA; and (3) 10 manually recorded points of the exhaled breath’s relative humidity (RH) from a digital psychrometer. These RH measurements were carefully taken at points where the subjects exhaled. 

The captured RR data from the Wi-Fi sensor were guided using a metronome, instructing the subject’s breathing. After the measurements, the captured data were processed, filtered, and analysed further. At the same time, RH measurements were taken first using the psychrometer, and then using the UWB antenna, at each 30 s interval for an exhaled breath. This enabled a preliminary investigation of the correlation between the captured exhaled breath data from the UWB antenna and the data obtained from the digital psychrometer. Finally, both datasets were compared and analysed to distinguish a correlation between RR and exhaled breath. 

For capturing the exhaled breath data, a VNA was used to capture data from the UWB antenna. The experiments were conducted using a host PC that sent commands to the VNA to start generating signals and capturing (or S_11_) data from the ports using built-in software and a GPIB. The VNA used for the experiments was an HP 8753 with a frequency range of 300 kHz to 3 GHz and a dynamic range of up to 100 dB. The RF sensors were connected to the 50-ohm ports on the VNA using SMA cables. The UWB antenna was placed 75 cm in front of the subject, as shown in Figure 3. This distance was set to ensure that the UWB antenna could detect the exhaled breaths of the subject while not interfering with the performance of the Wi-Fi antennas. 

For each breathing scenario, 10 measurements were obtained by the VNA for each subject, totalling 60 data points. In addition, a digital psychrometer (Protmex PT6508) was used to measure the relative humidity percentage in the air exhaled from the subject’s mouth. For each breathing rate, a total of 10 measurements were recorded using the psychrometer for each subject. This value was then compared to the relative humidity of the environment, which was found to have an RH of 45% at a temperature of 17 °C. The change in RH from the psychrometer was then compared to the captured S_11_ data from the UWB antenna in order to determine whether a correlation existed between the amount of water vapour in the exhaled air and the captured data from the antennas.

Operating at the same time as the UWB antenna, the RR of the subjects was measured using off-the-shelf ESP32 microcontrollers. The receiver collected the CSI data using the esp32-csi-tool [37], with a packet transmission rate set to 120 packets per second and timestamped at the receiver with UNIX time. Regarding the sensor placement, the TX and RX were set 1 m apart, at 1 m height from the ground level, and the participant was seated about 0.5 m away from the mid-TX-RX LOS. Furthermore, a metronome was used as a ground-truth device to control the subject’s breathing rate. A metronome program was run in the background to double the rate of intended RR, and the participant was asked to breathe in and breathe out, corresponding to alternative beats.

## 3. Results

### 3.1. Respiratory Rate Data Captured from Wi-Fi Antennas

The respiratory rate was taken as an independent variable in this investigation and was measured using the unobtrusive Wi-Fi sensors. A metronome controlled the RR, and the extracted RR from Wi-Fi sensors was noted against the ground-truth RR. Regarding the signal pre-processing, the CSI amplitude data from the Wi-Fi sensors were downsampled and filtered using the discrete wavelet transform with a ‘db4’ wavelet for RR corresponding to the range of expected human resting RR of 10 to 37 BPM [38]. Subsequently, the second principal component was extracted, since it contained more of the respiration waveform. The RR value was then determined from the peak of the power spectral density of the second principal component. As shown in Table 2, the Wi-Fi sensor successfully obtained the expected RR and, thus, could be used to measure the RR unobtrusively as an independent variable for examining the effect of RR on exhaled relative humidity. There was a slight difference of 0.5 BPM for Subject 2 during the 12 BPM case, which may have been due to unfiltered noise in the captured signal. 

### 3.2. Exhaled Breath Data Captured by the Digital Psychrometer 

Before conducting the experiments on the exhaled breath, the RH and temperature of the room were measured using the digital psychrometer. These were found to be approximately 38.4%RH and 20.2 °C, respectively. Each subject then performed the breathing exercises using a metronome in order to control their breathing rates to the desired levels. Each subject then used the digital psychrometer to measure the RH from the exhaled breath at each breathing rate. A total of 10 readings were captured using the psychrometer and averaged. 

**Figure 4:**
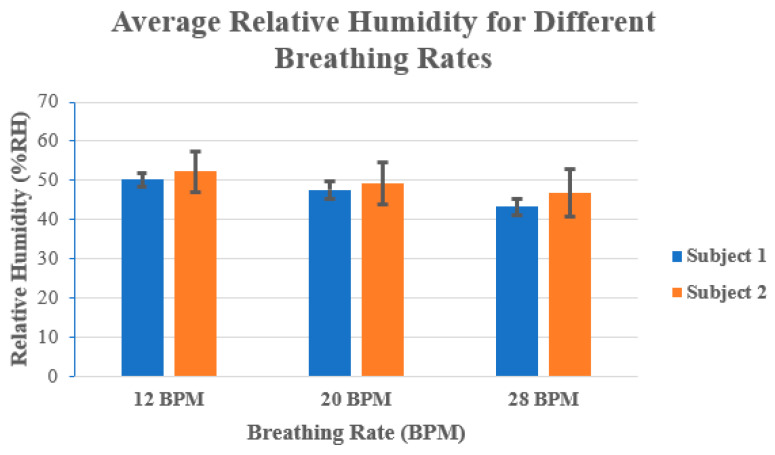
Average relative humidity (in %RH) for both subjects at 12 BPM, 20 BPM, and 28 BPM.

Figure 4 shows the average % RH values for each subject at different breathing rates. For example, it can be seen in Figure 4 that at 12 BPM the RH was larger, and it decreased when the number of BPM increased. At 12 BPM, both subjects had more than 50% RH, while at 28 BPM both subjects had an approximately 7 to 8% decrease in the average RH. This can be attributed to the amount of exhaled air. At lower breathing rates, there is a larger volume of exhaled air compared to higher breathing rates, where the volume of exhaled air is much smaller.

In addition, the exhaled air at lower breathing rates also contains a large amount of moisture from the lungs, airways, and mouth, which contributes to the increase in RH.

### 3.3. Exhaled Breath Data Captured from the UWB Antenna

Next, the captured data from the UWB antenna were processed and filtered in MATLAB in order to remove any significant noise from the readings. In addition, because the UWB antenna operates between 0.75 to 3 GHz, it was decided to remove data captured at frequencies below 0.75 GHz, as these were outside its operating frequency range and would not be useful. Finally, to understand the effect of exhaled breaths on S_11_ values, the change in S_11_ was calculated for each case (i.e., 12 BPM, 20 BPM, and 28 BPM) with the measured S_11_ of the antenna in free space (subject in front of the antenna, but not breathing towards it). The mean value for the change in S_11_ was calculated for each case and compared with those obtained from the digital psychrometer. Table 3 shows the average change in S_11_ for each case, along with the change in %RH value that was captured with the digital psychrometer. 

For both subjects, we can see an increase in S_11_ of more than 230% at the lower breathing rate of 12 BPM. Conversely, at the higher breathing rate of 28 BPM, the change in S_11_ increased by only 28–35% for both subjects. This finding, along with the %RH trend, can be attributed to the larger concentration of water vapour that was present in the exhaled breath at lower breathing rates. As an additional result of this increase in water vapour concentration in the air, it is no surprise that the area surrounding the mouth, at the time of exhalation, would have a larger increase in its dielectric properties, namely, the relative permittivity. It is therefore because of this increase in the dielectric properties at lower breathing rates that the propagated RF waves from the antenna were more easily absorbed, presenting a significantly larger loss in the reflected signals.

## 4. Discussion

### Comparison of Results Captured from the Wi-Fi Sensor and UWB Antenna 

**Figure 5:**
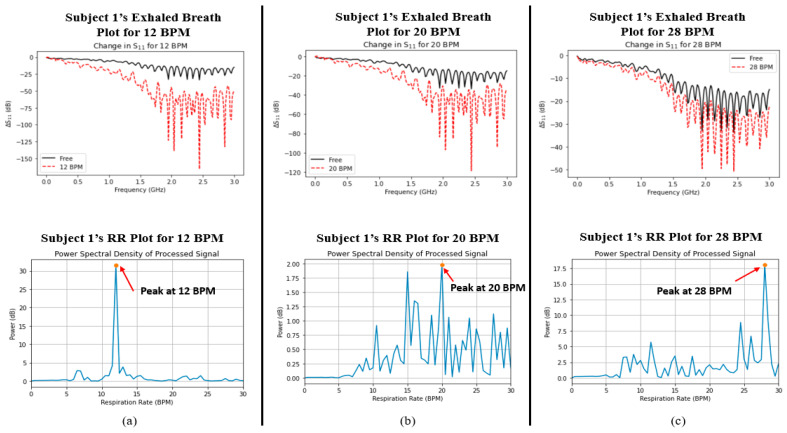
Graphs depicting the measured water vapour content in exhaled breath (indicated by ΔS_11_, top row) and respiratory rate (indicated by power spectral density, bottom row) for Subject 1 at (**a**) 12 BPM, (**b**) 20 BPM, and (**c**) 28 BPM.

**Figure 6:**
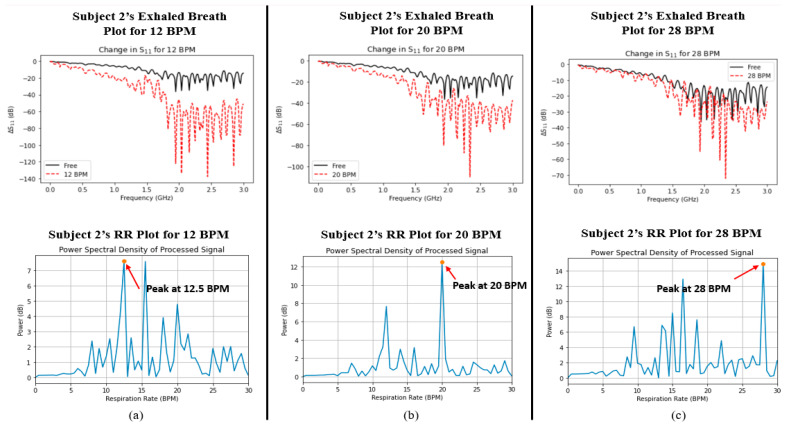
Graphs depicting the measured water vapour content in exhaled breath (indicated by ΔS11, top row) and respiratory rate (indicated by power spectral density, bottom row) for Subject 2 at (**a**) 12 BPM, (**b**) 20 BPM, and (**c**) 28 BPM.

Finally, a comparison was made between the captured signals for both parameters in order to determine whether the two variables were correlated with one another. Figure 5 and Figure 6 show the plots of data captured for both parameters (i.e., exhaled breath and RR) in each case for Subjects 1 and 2, respectively. Although there were nearby peaks in the PSD analysis, we have previously tested the reliability of our Wi-Fi CSI sensing system and observed that it operates with acceptable reliability for care monitoring purposes [30].

The ΔS_11_ plots in Figure 5 and Figure 6 show a very interesting trend. Like the average %RH that was shown in Figure 4 earlier, there exists the same trend, namely, that the change in S_11_ greatly increases as the RR decreases. In addition, the power spectral density plots for both subjects also show the peak power of the processed signal occurring at the correct times associated with the RR of the subject. In order to determine whether a relationship existed between the two datasets (i.e., S_11_ and power spectral density/BPM), the Pearson correlation coefficient (*r*) was captured for each case using MATLAB. Table 4 shows the calculated Pearson correlation coefficient for RR (*r_RR_*) and exhaled breath (*r_S_*_11_) for each case.

Since the values of *r_RR_* and *r_S_*_11_ were both below 0.4, this indicates a very weak positive correlation between RR and water vapour from the exhaled breath. Hence, there is no mutual relationship between the two parameters; they are independent of one another.

## 5. Conclusions

This study investigated the use of sensors and antennas to unobtrusively detect two distinct breathing parameters: respiratory rate, and the changes in the water vapour volume in the exhaled air. Previous studies have focused on monitoring respiratory rate and tidal volume as indicators of breathing abnormalities for the ageing population. The research described in this study provides a novel, inexpensive, and effective approach for the unobtrusive and noninvasive monitoring of breathing and relative humidity amongst the ageing population. Most importantly, it is also secure and private to the individual, as it does not rely on the use of cameras to monitor a person’s breathing. In addition, using data from both of these types of sensors and antennas, combined with sophisticated machine learning and AI algorithms, can provide a more holistic view of what respiratory issues a person is facing and, therefore, ensure that timely care is provided to them. 

The authors aim to expand this work further with the promising results obtained in this initial proof-of-concept. Future work in this area will look at involving people from the patient and public committee to provide feedback on the design and placement of the antennas, as well as whether there are other considerations to take. In addition, planned future work will look to optimise the antennas and data processing algorithms further to improve the accuracy and efficiency of the antennas’ performance. Future experiments of scenarios such as respiratory rate and hydration during sleep, as well as post-physical activity, will be conducted to expand the system’s applicability for indoor health management. Finally, once the required research ethics are determined, the authors aim to conduct more experiments in a pre-clinical setting, where volunteers from different age groups, and with different respiratory health issues, can participate in order to validate the effectiveness and reliability of the antennas in a more realistic setup.

## Figures and Tables

**Table 1:** Geometric dimensions of ultra-wideband antenna.

Symbol	Parameter Name	Value (mm)
*L*	Antenna Length	80
*W*	Antenna Width	35
*h*	Substrate Thickness	0.2
*w*	CPW Gap	0.65
*f_w_*	CPW Feed-Line Width	5.714
*f_l_*	CPW Feed-Line Length	22.857
*g_w_*	CPW Ground Length	50.29

**Table 2:** Measured respiration rate.

RR (Ground Truth)	RR (Wi-Fi Sensor, Subject 1)	RR (Wi-Fi Sensor, Subject 2)
12 BPM	12 BPM	12.5 BPM
20 BPM	20 BPM	20 BPM
28 BPM	28 BPM	28 BPM

**Table 3:** Comparison of the changes in S_11_ and relative humidity for exhaled breath.

	Subject 1		Subject 2	
CASE	ΔS_11_ (%)	ΔRH (%)	ΔS_11_ (%)	ΔRH (%)
12 BPM	252.18 ± 20.87	28.75 ± 1.719	233.89 ± 22.43	34.24 ± 5.15
20 BPM	142.64 ± 12.65	22.23 ± 2.22	143.03 ± 14.01	26.56 ± 5.23
28 BPM	50.03 ± 5.63	10.93 ± 2.02	50.22 ± 6.94	20.21 ± 5.89

**Table 4:** Measured respiratory rate for each subject.

Case	*r_RR_*	*r_S11_*
12 BPM	0.2256	0.2256
20 BPM	0.3206	0.3206
28 BPM	0.2892	0.2892

## Data Availability

Data are contained within the article.
